# m^6^A Methylation Mediated Autophagy and Nucleotide-Binding Oligomerization Domain-like Receptors Signaling Pathway Provides New Insight into the Mitigation of Oxidative Damage by Mulberry Leaf Polysaccharides

**DOI:** 10.3390/ijms26094345

**Published:** 2025-05-02

**Authors:** Wenqiang Jiang, Yan Lin, Linjie Qian, Siyue Lu, Zhengyan Gu, Xianping Ge, Linghong Miao

**Affiliations:** 1 Key Laboratory of Freshwater Fisheries and Germplasm Resources Utilization, Ministry of Agriculture and Rural Affairs, Freshwater Fisheries Research Center, Chinese Academy of Fishery Sciences, Wuxi 214081, China; jiangwenqiang@ffrc.cn (W.J.); liny@ffrc.cn (Y.L.); lusiyue@ffrc.cn (S.L.); guzhengyan@ffrc.cn (Z.G.); gexp@ffrc.cn (X.G.); 2 Wuxi Fisheries College, Nanjing Agricultural University, Wuxi 214081, China; 2022213003@stu.njau.edu.cn

**Keywords:** *Megalobrama amblycephala*, mulberry leaf polysaccharides, m^6^A methylation, transcriptome, autophagy

## Abstract

m^6^A methylation modification is an important genetic modification involved in biological processes such as sexual maturation, antibacterial, and antiviral in aquatic animals. However, few studies have been conducted in aquatic animals on the relationship between m^6^A methylation modification and autophagy-inflammation induced by lipid metabolism disorders. In the present study, a high-fat (HF) group and HF-MLP group (1 g mulberry leaf polysaccharides (MLPs)/1 kg HF diet) were set up. The mid-hind intestines of *Megalobrama amblycephala* juveniles from the two groups were collected for MeRIP-seq and RNA-seq after an 8-week feeding trial. The m^6^A peaks in the HF and HF-MLP groups were mainly enriched in the 3′ Untranslated Region (3′UTR), Stop codon, and coding sequence (CDS) region. Compared with the HF group, the m^6^A peaks in the HF-MLP group were shifted toward the 5′UTR region. ‘RRACH’ was the common m^6^A methylation motif in the HF and HF-MLP groups. Methyltransferase *mettl14* and *wtap* expression in the intestines of the HF-MLP group were significantly higher compared with the HF group (*p* < 0.05). A total of 21 differentially expressed genes(DEGs) with different peaks were screened by the combined MeRIP-seq and RNA-seq analysis. Kyoto encyclopedia of genes and genomes (KEGG) enrichment analysis enriched BCL2 interacting protein 3 (*bnip3*) to autophagy–animal and mitophagy–animal signaling pathways, etc., and nucleotide-binding domain leucine-rich repeat protein 1 (*nlrp1*) was enriched to the Nucleotide-binding oligomerization domain (NOD)-like receptor signaling pathway. Combined MeRIP-seq and RNA-seq analysis indicated that the expression pattern of *bnip3* was hyper-up and that of *nlrp1* was hyper-down. Gene Set Enrichment Analysis (GSEA) analysis confirmed that the intestinal genes of HF-MLP group positively regulate lysosomal and autophagy–animal signaling pathways. In the present study, we demonstrated that m^6^A methylation modification plays a role in regulating autophagy-inflammatory responses induced by HF diets by MLPs, and further explored the molecular mechanisms by which MLPs work from the epigenetic perspective.

## 1. Introduction

*Megalobrama amblycephala* belongs to Cypriniformes, Cyprinidae, and *Megalobrama* (Dybowsky, 1792). The total farmed production of bream in China was 767,000 tons in 2023 (more than 90% of which was accounted for by *M. amblycephala*), which ranked eighth in China’s freshwater farmed fish production [1]. With the rapid development of the intensive aquaculture model in recent years, the farming production of *M. amblycephala* has been increasing, while its food safety problems have also become increasingly prominent [2]. High-fat diets have been used in intensive aquaculture models for their protein-sparing and growth-promoting properties to decrease production costs and enhance aquaculture production [3,4]. However, *M. amblycephala* chronically feeding on high-fat diets induces excessive lipid deposition, raises lipid peroxidation levels, inhibits autophagy, and induces oxidative stress and inflammation [5,6,7]. It has been found that the active substances extracted from plant resources with green, efficient, and non-polluting characteristics can improve the growth performance and fat utilization of fish and eliminate the adverse effects of a high−fat diet on fish [8,9].

Plant polysaccharides are important natural macromolecules made from glucose, fructose, galactose, arabinose, and other monosaccharides linked by α− or β-glycosidic bonds in plant cells [10]. The supplementation of plant polysaccharides in high-fat diets for aquatic animals promotes lipolysis and alleviates inflammation and oxidative stress caused by lipid metabolism disorders; for example, 2–4 g/kg sea buckthorn polysaccharide in the *Danio rerio* diet [11], 10 g/kg *Lycium barbarum* polysaccharide in the hybrid grouper (*Epinephelus fuscoguttatus♀ × Epinephelus lanceolatus♂*) diet [12], and 0.8 g/kg *Citrus maxima* polysaccharide in the hybrid grouper (*E. fuscoguttatus♀ × E. lanceolatus♂*) diet [13]. The area of mulberry plantations in China was 796,700 hm^2^ in 2021 [14], and the annual production of mulberry leaves exceeds 15 million tons [15]. Mulberry leaf polysaccharides (MLPs) are important active substances found in mulberry leaf, exhibiting various biological activities such as hypoglycemic, hypolipidemic, immune-boosting, anti-bacterial, anti-oxidant, and anti-aging effects, etc. [2,16,17,18,19]. MLPs also have favorable effects on improving the growth and development of the animal, immune function, and the quality of animal products in 14-day-old chickens and immunosuppressed mice in research [18,20]. MLPs supplementation improves metabolic disorders, repairs histopathological damage, and regulates intestinal flora in the high-fat diet-induced obese mouse model [21,22,23], yet little research has been conducted in aquaculture.

Autophagy is a highly conserved intracellular degradation pathway in eukaryotes which participates in biological processes related to cellular quality control, metabolism, and innate/acquired immunity [24]. Autophagy flux is affected by chronic high-fat diets or other causes of overnutrition, potentially inhibiting autophagy [25]. Infections, autoimmune disorders, and metabolic disorders may result when autophagy is dysfunctional [26]. Dietary high-fat diets in yellow catfish (*Pelteobagrus fulvidraco*) inhibited the formation of hepatic autolysosome. In vitro studies have shown that the formation of autolysosome in *P. fulvidraco* hepatocytes was significantly inhibited by incubation with palmitic acid [27]. Ultrastructural observations show that liver mitochondrial biogenesis and mitophagy were inhibited when spotted seabass (*Lateolabrax maculatus*) fed a high-fat diet [28]. Inflammasomes are multiprotein complexes that are activated and assembled in response to cellular stress or infection [29]. Research on the nucleotide-binding oligomerization domain-like receptors (NLR) family, an intracellular pattern recognition receptor with similar function and structure, such as NLRP1 and NLRP3, has centered on the relationship between autophagy and NLRP1/NLRP3 to explore the interactions between autophagy and inflammasomes [30,31]. NLRP3 inflammasome was activated by the inhibition of podocyte autophagy in high-fat diet-induced diabetic nephropathy mice, and silencing of NLRP3 effectively restored autophagy in podocytes, suggesting that NLRP3 is a negative regulator of autophagy [32]. Caspase-1 activation induced by NLRP1 inflammatory vesicle assembly leads to the release of pro-inflammatory cytokines interleukin 1β and interleukin 18 [33]. NLRP1 and NLRP3 mediate lipopolysaccharide-induced apoptosis in fibroblast-like synoviocytes, and inhibition of *nlrp1* and *nlrp3* markedly suppressed the expression of apoptosis-related cytokines [34].

Previous studies revealed that epigenetic mechanisms regulate the expression of autophagy genes, and that epigenetic mechanisms also affect autophagy by influencing the expression of genes upstream and downstream of autophagy [35]. Meanwhile, the *nlrp1* expression is also mediated by m^6^A methylation modification [36]. Dietary levels of carbohydrates, vitamins, and other substances have been found to be strongly associated with epigenetic modifications in aquatic animals [37,38,39]. Briefly, nutritional factors affect gene expression and their regulation of biological processes through epigenetic modifications at the molecular level [38]. High-fat diets lead to extensive gene promoter methylation alterations in humans and mice, affecting organ development and function [40,41]. In this study, MLPs were supplemented to high-fat diets for *M. amblycephala*, aiming to establish an epigenetic regulatory network of the MLPs-regulated autophagy and NOD-like receptor signaling pathway in mitigating high-fat diet-induced intestinal damage by integrating the intestinal transcriptome and m^6^A methylation sequencing.

## 2. Results

### 2.1. m^6^A Methylation Modification in Intestines of HF and HF-MLP Groups

**Figure 1:**
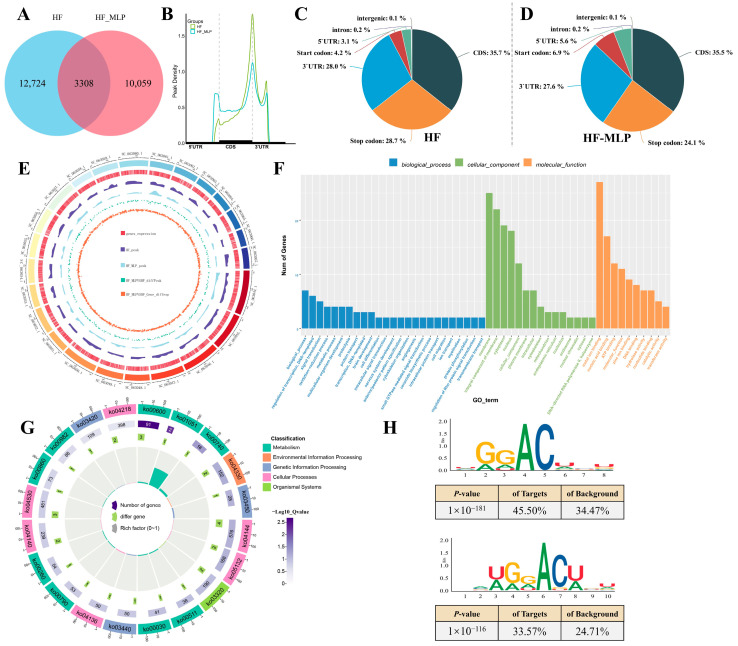
MLPs altered m^6^A modification in the intestines of HF and HF-MLP groups. (**A**) Classification of methylated genes in the intestines of the HF and HF-MLP groups. (**B**–**D**) Peak density analysis and classification of m^6^A methylation modification sites in intestinal mRNA of the HF and HF-MLP groups. (**E**) Circos plot of the levels of m^6^A peaks and expression abundance on 24 chromosomes in the HF and HF-MLP groups. (**F**) GO categorization analysis of genes with differentially methylated m^6^A peaks. (**G**) KEGG pathway analysis of genes with differential methylated m^6^A peaks. (**H**) The same motif which was identified in the intestines of the HF and HF-MLP groups.

High-throughput sequencing results of the intestinal IP (MeRIP-seq) and input (RNA-seq) libraries showed that more than 82.0% of the clean data mapped the reference genome of *M. amblycephala*, with excellent concordance between different replicates, Q30 > 94.4% and Q20 > 98.0% (Tables S1 and S2). Venn diagram analysis of the HF and HF-MLP groups revealed 3308 common peaks, and 12,724 unique peaks to the HF group as well as 10,059 unique peaks to the HF-MLP group (Figure 1A). In terms of the m^6^A peak distribution, the m^6^A peaks were shifted with MLPs supplementation, and the m^6^A peak density in the 5′UTR region of the HF-MLP group increased by 2.5% compared with that of the HF group (Figure 1B–D). Briefly, 35.7%, 28.0%, and 3.1% of the m^6^A peaks were located in the CDS, 3′UTR, and 5′UTR regions in the HF group, while in the HF-MLP group, these percentages were 35.5%, 27.6%, and 5.6%, respectively. Over 87.9% m^6^A peaks in HF group and 96.2% m^6^A peaks in HF-MLP group were mapped to the CDS region of the reference genome (Table S3, Figure S1). Gene expression and genomic distribution of m^6^A peaks were almost identical on the 24 major chromosomes of the reference genome of *M. amblycephala* (Figure 1E).

In Gene Ontology (GO) categorization analysis, the category with the greatest number of differential m^6^A peaks was highly enriched in the biological process (biological process ontology), membrane (cellular component ontology), and metal ion binding (molecular function ontology) (Figure 1F, Table S4). Kyoto Encyclopedia of Genes and Genomes (KEGG) enrichment analysis significantly enriched 35 pathways (Figure 1G, Table S5). The top 10 KEGG pathways (based on *p*-value) that differential m^6^A peaks most significantly enriched were sphingolipid metabolism (ko00600), biosynthesis of ansamycins (ko01051), riboflavin metabolism (ko00740), notch signaling pathway (ko04330), non-homologous end-joining (ko03450), endocytosis (ko04144), salmonella infection (ko05132), PPAR signaling pathway (ko03320), other glycan degradation (ko00511), and the pentose phosphate pathway (ko00030).

Furthermore, we found that the RDACW motif was identified as highly enriched within the m^6^A site in the HF and HF-MLP groups by motif analysis (Figure 1H). According to the abbreviated base symbols correspondence table, “A/G” is denoted by “R”, “U/A/G” is denoted by “D”, “A/U” is denoted by “W”.

### 2.2. Identification of DEGs in Intestinal of HF and HF-MLP Groups

**Figure 2:**
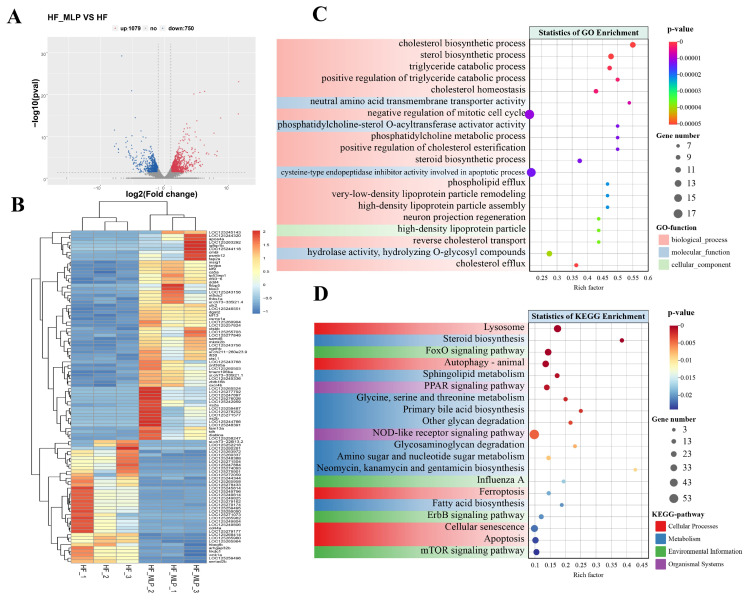
MLPs affected mRNA expression in the intestines of HF and HF-MLP groups. (**A**) Volcano plots were used to characterize significantly different genes based on Log_2_ (Fold change). (**B**) Heat map of transcriptome profile for HF and HF-MLP groups. Rows represent biological replicates and columns represent individual genes. Transcripts with higher expression levels in a sample are displayed in red and yellow, whereas those with lower expression levels are displayed in blue. (**C**) Enriched GO terms performed with Goatools package (https://github.com/tanghaibao/goatools) for DEGs between the HF and HF-MLP groups. (**D**) Enriched KEGG pathways performed with KOBAS 3.0 (http://bioinfo.org/kobas) for DEGs between HF and HF-MLP groups.

Transcriptome sequencing of input libraries constructed in the HF and HF-MLP groups showed that the number of DEGs in the HF-MLP group vs. the HF group was 1829, of which 1079 DEGs were up-regulated and 750 DEGs were down-regulated (Figure 2A). The top 100 DEGs with significant differences were subjected to expression pattern clustering analysis (Table S6). Figure 2B clearly presents that the DEGs within the same group have similar expression patterns and expression levels, while the differential genes between different groups show significant separation effects.

GO enrichment analysis significantly enriched 2695 functions (Figure 2C, Table S7). The top 10 GO functions (based on *p*-value) that DEGs most significantly enriched were cholesterol biosynthetic process (GO:0006695), sterol biosynthetic process (GO:0016126), triglyceride catabolic process (GO:0019433), positive regulation of triglyceride catabolic process (GO:0010898), cholesterol homeostasis (GO:0042632), neutral amino acid transmembrane transporter activity (GO:0015175), negative regulation of mitotic cell cycle (GO:0045930), phosphatidylcholine-sterol O-acyltransferase activator activity (GO:0060228), phosphatidylcholine metabolic process (GO:0046470), and positive regulation of cholesterol esterification (GO:0010873).

KEGG enrichment analysis significantly enriched 197 pathways (Figure 2D, Table S8). The top 10 KEGG pathways (based on *p*-value) that DEGs most significantly enriched were lysosome (ko04142), steroid biosynthesis (ko00100), FoxO signaling pathway (ko04068), autophagy–animal (ko04140), sphingolipid metabolism (ko00600), PPAR signaling pathway (ko03320), glycine, serine and threonine metabolism (ko00260), primary bile acid biosynthesis (ko00120), other glycan degradation (ko00511), and NOD-like receptor signaling pathway (ko04621).The statistical information on the autophagy–animal and NOD-like receptor signaling pathway-related DEGs in the intestines of the HF-MLP group compared to the HF group was shown in Table 1.

### 2.3. Assessment of the m^6^A-Modified Gene Transcription in Intestinal of HF and HF-MLP Groups

**Figure 3:**
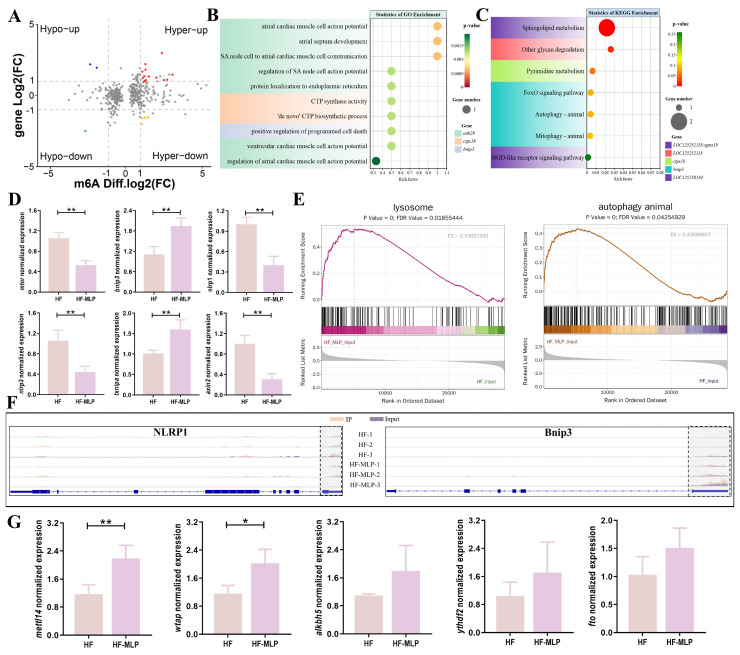
Integrating analysis of differentially modified m^6^A methylation and expressed genes in the intestines of HF and HF-MLP groups. (**A**) Four-quadrant graph representing the relationship between m^6^A methylation and gene expression. (**B**) GO analysis of genes with differential methylated m^6^A peaks and differential expression. (**C**) KEGG analysis of genes with differential methylated m^6^A peaks and differential expression. (**D**) Relative transcript levels of key genes (qRT-PCR validation, *n* = 8). ** indicates an extremely significant difference (*p* < 0.01; Independent sample *t*-test). (**E**) GSEA analysis for the lysosome and autophagy–animal pathway. (**F**) Peak abundance of m^6^A (IP) and expression (input) in *nlrp1* and *bnip3* in intestines of HF and HF-MLP groups. The dashed box represents the 3′UTR region of the gene. (**G**) Relative gene expression levels of methylase-related genes (*n* = 8). * indicates a significant difference (*p* < 0.05; Independent sample *t*-test); ** indicates an extremely significant difference (*p* < 0.01; Independent sample *t*-test).

Figure 3A shows a four-quadrant graph of genes with significant differences in both m^6^A methylation and genes expression (Threshold: **|**Log_2_(Fc)**|** > 1). The expression pattern of 13 genes was m^6^A hypermethylation and gene expression was up-regulated (Hyper-up). The expression pattern of four genes was m^6^A hypermethylation and gene expression was down-regulated (Hyper-down). The expression pattern of two genes was m^6^A hypomethylation and gene expression was up-regulated (Hypo-up). The expression pattern of two genes was m^6^A hypomethylation and gene expression was down-regulated (Hypo-down) (Table 2).

GO enrichment analysis significantly enriched 107 functions (Figure 3B, Table S9). The top 10 GO functions (based on *p*-value) that m^6^A-modified DEGs most significantly enriched were atrial cardiac muscle cell action potential (GO:0086014), atrial septum development (GO:0003283), SA node cell to atrial cardiac muscle cell communication (GO:0086070), regulation of SA node cell action potential (GO:0098907), protein localization to the endoplasmic reticulum (GO:0070972), CTP synthase activity (GO:0003883), ‘de novo’ CTP biosynthetic process (GO:0044210), positive regulation of programmed cell death (GO:0043068), ventricular cardiac muscle cell action potential (GO:0086005), and regulation of atrial cardiac muscle cell action potential (GO:0098910). KEGG enrichment analysis significantly enriched seven pathways (Figure 3C, Table S10), sphingolipid metabolism (ko00600), other glycan degradation (ko00511), pyrimidine metabolism (ko00240), FoxO signaling pathway (ko04068), autophagy–animal (ko04140), mitophagy–animal (ko04137), and NOD-like receptor signaling pathway (ko04621).

Genes related to the autophagy–animal (*mtor*, *bnip3*), NOD-like receptor signaling pathway (*txnipa*, *nlrp1*, *nlrp3*), and Wnt signaling pathway (*axin2*) were randomly selected for verification (Figure 3D). All the candidate genes verified by qRT-PCR were identical to the results of transcriptome sequencing, indicating that the sequencing results were credible (Figure 3D, Table S11). GSEA confirmed that the intestinal genes of the HF-MLP group positively regulate lysosomal and autophagy–animal signaling pathways (Figure 3E).

Figure 3F exhibits the m^6^A peak expression patterns of two representative genes in the autophagy–animal signaling pathway (*bnip3*) and NOD-like receptor signaling pathway (*nlrp1*). *Bnip3* and *nlrp1* undergo hypermethylation in the 3′UTR region and cause changes in gene expression. In details, the expression of *nlrp1* was significantly down-regulated in the HF-MLP group versus the HF group, and the *bnip3* expression was significantly up-regulated in the HF-MLP group than in the HF group (|Diffgene.Log_2_(Fc)| > 1).

Expression of m^6^A methylation modification-related enzymes (*mettl14*, *wtap*, *ythdf2*, *fto,* and *alkbh5*) was detected by qRT-PCR. Notably, the changes in *wtap* and *mettl14* were more significant compared to several other key genes (Figure 3G). Noticeable increases in *mettl14* and *wtap* expression were exhibited in the HF-MLP group compared to the HF group (*p <* 0.05). The expression of *ythdf2*, *fto*, and *alkbh5* were not affected by dietary MLPs supplementation (*p* > 0.05).

## 3. Discussion

m^6^A methylation modification, the most abundantly expressed form of epigenetic regulation, is a dynamically reversible process [42]. m^6^A methylation genomics could be co-analyzed with metabolomics for a more comprehensive understanding of gene function. Epigenetic changes attributed to diet-induced m^6^A methylation modification were found to mediate phenotypic plasticity in humans and mammals [43,44,45]. However, researchers in the fisheries field have primarily focused on the dynamic m^6^A methylation modification characteristics of aquatic animals in different physiological states [46,47].

In the present study, the m^6^A modification profile of the *M. amblycephala* intestines was first characterized, and the m^6^A methylation modification sites of intestinal mRNAs were enriched near the 3′UTR, stop codon, and CDS region. Interestingly, m^6^A methyla-tion modifications of mRNAs were found to show differences in spatial distribution de-pending on the species. The m^6^A methylation modifications were mainly enriched in the exon region, stop codon region, and 3′UTR region in humans [43], rats [44], and finishing pigs [45] suggesting that the overall distributions of m^6^A methylation modification sites in the human, mammal, and *M. amblycephala* are similar [48]. By contrast, studies in plants have revealed that m^6^A methylation modification sites are enriched not only in the stop codon region and the 3′UTR region, but also around the start codon and the 5′UTR region [49,50]. The proportion of m^6^A peaks in the 5′UTR region and stop codon region in the HF-MLP group was higher than the proportion of m^6^A peaks in the HF group, suggesting that the distribution of m^6^A peaks may be affected by MLPs supplementation. Furthermore, the localization of m^6^A peaks shifted to the 5′UTR and CDS regions after MLP supplementation in the present study. The dynamic distribution of m^6^A methylation modifications is highly responsive to regulating animals’ physiological conditions to accommodate various changes in the external environment [51]. Consistent with previous studies, the m^6^A peak of the mRNA was also found to move toward the 5′UTR region following changes in the external environment in humans, large yellow croaker (*Larimichthys crocea*), and *Arabidopsis* [47,51,52]. Annotation analysis showed that the common base sequence of the different methylation modification sites was RDACW in the intestines of the HF and HF-MLP groups. The distribution characteristics of RDACW are consistent with the common m^6^A motif pattern RRACH, indicating that a highly conserved type of m^6^A methylation modification sites, which further confirms that the function of m^6^A methylation modification is indeed present in *M. amblycephala* intestine [53,54].

m^6^A methylation modification plays an essential regulatory role in animal reproduction, growth development, and immunization. m^6^A methylation modification is undertaken by writers (*mettl3*, *mettl14*, *wtap*, etc.), erasers (*fto* and *alkbh5*), and readers (*ythdc1*, *ythdf2*, *ythdf3*, etc.) for the addition, deletion, or recognition of m^6^A methylation modifications. In the present study, intestinal *mettl14* and *wtap* (writers) expression was significantly higher in the HF-MLP group compared with the HF group. Conserved heterodimers formed by methyltransferase 13 (METTL3) and METTL14 are recruited by wtap to form a complex that mediates the m^6^A methylation modification process [55]. Overexpression/knockdown of METTL14 mediates osteogenic differentiation capacity of bone marrow mesenchymal stem cells by activating/suppressing autophagy through m^6^A methylation modification of beclin-1 expression [56]. METTL14 overexpression activates the autophagy pathway in the mitochondria of I/R cardiomyocytes to ameliorate cardiomyocytes injury [57]. Hypotheses based on classical physiology and biochemistry suggest that epigenetic regulation is a potential factor in dietary modification of animal phenotype. Betaine potentiates lipolysis in finishing pigs feeding on low-energy diets by up-regulating the expression of writer genes/proteins (*mettl3*, *mettl14*, *wtap*) [45]. Notoginsenoside R1 repairs DNA damage in skin under UV irradiation by up-regulating wtap expression in skin keratinocytes [58].

m^6^A methylation modification as a pivotal mediator in response to dietary and external environmental changes has received mounting awareness [43,45,50]. In the present study, we analyzed and identified 21 DEGs (|DiffExp.gene.Log_2_(Fc)| > 1) with differential m^6^A Peak (|DiffPeak.Log_2_(Fc)| > 1). KEGG signaling pathway enrichment analysis showed that these DEGs were enriched in the autophagy–animal signaling pathway, and NOD-like receptor signaling pathway. In the autophagy–animal signaling pathway, *bnip3* was enriched, with an expression pattern of up-regulation of methylation levels and up-regulation of gene expression. A positive correlation between gene expression and methylation levels has been reported in most transcripts of *Arabidopsis* and human studies as compared with the expression pattern in which methylation levels are negatively correlated with gene expression [48,59]. Consistent with our research, up-regulation expression of *bnip3* in breast tumors via methylation in the 3′UTR plays a proapoptotic function [60]. *Astragalus* polysaccharide promotes cellular autophagy and ameliorates femoral head necrosis by elevating *bnip3* expression [61]. In the NOD-like receptor signaling pathway, *nlrp1* was enriched, with a classical expression pattern of up-regulation of methylation levels and down-regulation of gene expression. NLRs are pattern recognition receptors in the cytoplasm, and the activation of partial members (NLRP1, NLRP3, and NLRC4, etc.) leads to the assembly of inflammasome, which subsequently triggers the activation of inflammatory caspases [62]. The pro-inflammatory downstream effectors IL-1β and IL-18 were cleaved by enzymatically active Caspase-1 into their biologically active forms to induce inflammation [63]. *Antrodia camphorate* polysaccharides reduced reactive oxygen species (ROS) content to inhibit reduced nicotinamide adenine dinucleotide phosphate oxidase 2 (NOX2)-NLRP1 activation, thereby reducing inflammatory damage of cortical neurons [64]. The activation of NLRP1 inflammasome mediating inflammatory responses in aquatic animals has been reported in both zebrafish [63] and common carp (*Cyprinus carpio*) [65]. NLRP1 was found to play an essential role in the immune organs of bony fish, and its expression was up-regulated in common carp (*Cyprinus carpio*) treated with spring viremia of carp, *Edwardsiella tarda*, and *Aeromonas hydrophila* [65].

In the present study, KEGG analysis of DEGs in the transcriptome and DEGs with differential peaking following coanalysis revealed that the differential genes were enriched in the autophagy–animal and NOD-like receptor signaling pathways. Furthermore, GSEA analysis showed that the expression of major genes of autophagy–animal and lysosomal signaling pathways were up-regulated in the HF-MLP group. Autophagy, an important cellular mechanism in eukaryotic cells, degrades damaged organelles and macromolecules via lysosomes [24]. In aquatic animals such as *M. amblycephala* [6,66], zebrafish [67], and spotted seabass (*Lateolabrax maculatus*) [28], etc., it was found that high-fat diets down-regulated the expression of autophagy-related genes and inhibited autophagic flux. MLPs in this present study were heteropolysaccharides (201.4 KDa) mainly consisting of rhamnose, arabinose, and galactose, a structure with an extreme immune and antioxidant activity [2]. Plant polysaccharides were attenuated by high-fat diet-induced lipid accumulation, oxidative stress, and inflammation by activating autophagy [68,69,70]. It has been found that polysaccharides might play an immunoregulatory and inflammatory function by modifying the levels of non-coding RNA [71], histone modifications [72], etc. Tremella fuciformis polysaccharides attenuate lipopolysaccharide-induced inflammatory responses by down-regulating miR-155 expression in RAW264.7 cells that suppress the expression of protein kinase B (AKT), p38MAPK, and nuclear factor kappa-B (NF-κB) [73]. Stimulus factors such as pathogen/injury initiate the assembly of NLRP1/NLRP3 inflammasome to activate inflammasome [29]. Autophagy inhibits inflammatory responses by phagocytosis and degradation of inflammasome and downstream active components through endocytosis and phagocytosis [74]. Micheliolide mediates autophagy to degrade some components of the NLRP3 inflammasome, blocking inflammasome activation and attenuating pro-inflammatory cytokine release in radiation-damaged intestinal tissues [75]. Autophagy dysfunction results in blocked clearance of activated NLRP3 inflammasome to induce maturation and release of pro-inflammatory cytokines [29,32]. Moreover, activation of inflammasome-mediated IL-1β secretion aggravates intestinal ischemia-reperfusion injury by inhibiting autophagy in mice, whereas knockdown of NLRP3 reverses these effects [76].

## 4. Materials and Methods

### 4.1. MLPs

MLPs was obtained from Xi’an Ciyuan Biotech Co., Ltd., Xi’an, China. MLPs were heteropolysaccharides (201.4 KDa) mainly consisting of rhamnose, glucose, arabinose, and galactose. UV-Vis spectroscopy (Thermo Fisher, Cleveland, OH, USA) showed that the MLPs were extremely pure, with minimal protein and nucleic acid content. MLPs are pyranose rings polysaccharides linked by β-glycosidic bonds as revealed by FT-IR spectra (Thermo Fisher, Cleveland, OH, USA) [3].

### 4.2. Diets

Crushed ingredients (sieved through a 60-mesh) such as soybean meal, rapeseed meal, and cottonseed meal were mixed well according to the feed formula, with soybean oil and water added, and 2.0 mm sink pellets were made with an F-26 (II) granulator (South China University of Technology, Guangzhou, China). Two types of diets were made in our experiment: an HF diet (crude protein 31.7%, crude fat 14.8%) and an HF-MLP diet (1 g/kg of MLPs was added to the formula of HF diet to make HF-MLP diet). Diets dried in a cool dry place were packaged and stored at -20 °C until the feeding trial.

### 4.3. Fish and Feeding Trial

Experimental fish species was *M. amblycephala* ‘Huahai No.1’, which was supplied by the National *M. amblycephala* stock (Ezhou, China). Juveniles of homogeneous size (9.67 ± 0.23 g) were selected for the feeding trial, with 15 juveniles per aquarium in six aquariums (0.32 m^3^). The HF and HF-MLP groups were set up in the feeding trial, with three replicates in each group. *M. amblycephala* juveniles were fed with commercial feed (Tongwei Co., Ltd., Wuxi, China) during the domestication period. Fish were fed experimental diets three times a day (7:00, 12:00, and 17:00) since the completion of domestication. During the 8-week feeding trial, the conditions of the aquaculture water were kept constant with dissolved oxygen ≥ 6 mg/L, water temperature 26 °C~28 °C, ammonia nitrogen ≤ 0.05 mg/L, and pH 7.0 ± 0.1.

### 4.4. Sample Collection

Four fish were randomly selected from each aquarium while the feeding trial was ended, anesthetized with 100 mg/L tricaine methanesulfonate (MS-222) (Sigma, Saint Louis, MO, USA), and the emptied hind intestine was sampled on ice. Collected samples were snap-frozen in liquid nitrogen and stored at −80 ℃, awaiting MeRIP-seq, RNA-seq, real-time PCR analysis.

### 4.5. RNA Isolation, Library Construction, and Sequencing

RNA was extracted from the mid-hind intestine of the HF and HF-MLP groups using TRIzol reagent (Takara Co., Ltd., Dalian, China), with three replicates in each group, and downstream experiments were performed after quality control and integrity testing of the RNA. Poly (A) RNA specifically purified by Dynabeads Oligo (dT) (Thermo Fisher, Cleveland, OH, USA) was fragmented into small pieces at 86 °C for 7 min. The cleaved RNA fragments were incubated for 2 h at 4 °C with m^6^A-specific antibody (No. 202003, Synaptic Systems, Göttingen, Germany) in IP buffer. The IP RNA was reverse-transcribed to cDNA, which was next used to synthesize Ulabeled second-stranded DNAs. PCR was used to construct sequencing libraries following double-stranded digestion. At last, we performed paired-end sequencing (PE150) on an Illumina Novaseq™ 6000 platform (Biozeron Co., Ltd., Shanghai, China).

### 4.6. Bioinformatics Analysis

The clean data obtained after FastQC and RseQC from all IP samples and Input samples were analyzed. Reads were mapped to the reference genome of *M. amblycephala* using HISAT2 (http://daehwankimlab.github.io/hisat2). Peak calling and diff peak analysis were performed by R package exomePeak (https://www.bioconductor.org/packages/3.3/bioc/html/exomePeak.html). HOMER (http://homer.ucsd.edu/homer/motif) was used for motif analysis.

StringTie was used for quantified expression for all genes in the input libraries by calculating FPKM. The differentially expressed genes (DEGs) were selected with |Log_2_ (Fold change)| ≥ 1 and *p* value < 0.05 by R package edgeR.

### 4.7. Real-Time PCR Analysis (qRT-PCR)

Intestinal RNA (400 ng/μL, OD260/280 = 1.8–2.0) extracted using TRIzol reagent (Takara, Dalian, China) was synthesized to cDNA by PrimeScript RT reagent Kit (Takara, China). The qRT-PCR analysis was conducted on the CFX96 instrument (Bio-Rad, Hercules, CA, USA) with TB Green (Takara, Dalian, China) as the fluorescent dye. Primers for qRT-PCR are listed in Table 3. The relative expression of genes was determined by the 2^−∆∆CT^ method with *β−actin* as the reference gene.

### 4.8. Statistical Analysis

An independent sample *t*-test was performed to identify differences between the HF and HF-MLP groups using SPSS Version 20.0 software (SPSS Inc., Chicago, IL, USA) when data conformed to normality (Shapiro–Wilk test) and homogeneity of variance (Levene’s test). Values were expressed as the mean ± standard error. A statistically significant difference was considered at the *p* < 0.05.

## 5. Conclusions

**Figure 4:**
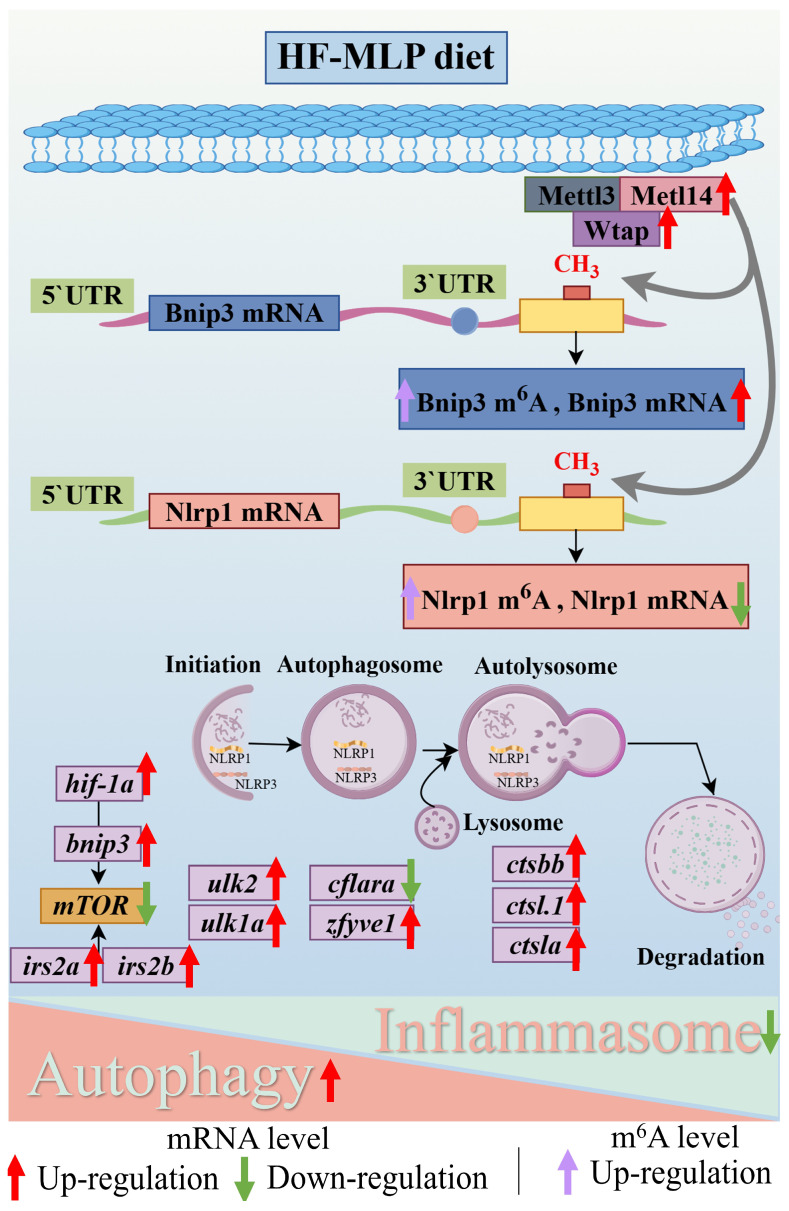
The epigenetic regulatory mechanisms by which the inclusion of mulberry leaf polysaccharides (MLPs) in high-fat diet affect intestinal health in *Megalobrama amblycephala*. Upward arrows indicate significant up-regulation in mRNA level or m^6^A level, and downward arrows indicate significant down-regulation in mRNA level.

In conclusion, diets of HF and HF-MLP induced a dynamic change in intestinal m^6^A methylation modification of *M. amblycephala* juveniles (Figure 4). Differential methylation modification of intestinal DEGs, mainly in the 3′UTR region, modulates the physiological status of the intestine in the *M. amblycephala* juveniles following the supplementation of MLPs. Combined MeRIP-seq and RNA-seq analyses identified key DEGs and pathways, autophagy–animal and mitophagy–animal signaling pathways (*bnip3*, Hyper-up), and NOD-like receptor signaling pathway (*nlrp1*, Hyper-down). This study provides strong support for diet (MLPs)-mediated epigenetic regulation to alter aquatic animal phenotypes.

## Figures and Tables

**Table 1:** Information statistics of autophagy–animal and NOD-like receptor signaling pathway-related DEGs in the intestines of HF-MLP group compared with HF group.

Predict Function	Gene Name	Transcript ID	Regulation	Log_2_FC	*p*-Value
Autophagy–animal	*ddit4*	XM_048171097.1	Up	4.33	0.00
	*hif1al2*	XM_048166513.1	Up	2.17	0.01
	*bnip3*	XM_048170148.1	Up	1.32	0.02
	*mtor*	XM_048210663.1	Down	−1.56	0.02
	*ulk2*	XM_048161082.1	Up	3.47	0.00
	*ulk1a*	XM_048209404.1	Up	1.23	0.00
	*irs2b*	XM_048193118.1	Up	4.79	0.00
	*irs2a*	XM_048197081.1	Up	3.62	0.00
	*zfyve1*	XM_048206589.1	Up	1.26	0.02
	*cflara*	XM_048173454.1	Down	−1.13	0.01
	*ctsbb*	XM_048207873.1	Up	2.91	0.00
	*ctsl.1*	XM_048191905.1	Up	3.14	0.00
	*ctsla*	XM_048189060.1	Up	2.11	0.00
NOD-like receptor signaling pathway	*mapk12b*	XM_048157097.1	Up	2.13	0.03
	*traf3*	XM_048191482.1	Down	−1.05	0.01
	*nlrc3*	XM_048175695.1	Up	1.94	0.01
	*nlrp3*	XM_048210961.1	Down	−3.50	0.00
	*nlrp12*	XM_048190599.1	Down	−3.12	0.00
	*nlrp1*	XM_048162530.1	Down	−1.59	0.00

**Table 2:** The expression pattern of m^6^A methylation-modified differentially expressed genes.

GeneName	MeRIP-Seq			RNA−Seq		Expression Pattern
	Peak Annotation	Diffpeak. Log_2_(Fc)	m^6^A Regulation	Diffgene. Log_2_(Fc)	Gene Regulation	
*wnk1a*	UTR3	−1.02	down	−1.90	down	Hypo-down
*loc125252147*	exonic	−2.44	down	−2.50	down	
*ank2b*	UTR3	−2.14	down	2.18	up	Hypo-up
*znhit6*	UTR3	−1.73	down	1.94	up	
*znf420*	UTR3	1.26	up	−1.56	down	Hyper-down
*loc125275941*	UTR3	1.06	up	−2.02	down	
*nlrp1*	UTR3	1.07	up	−1.59	down	
*map4*	exonic	1.45	up	−1.53	down	
*ubald2*	UTR3	2.32	up	2.97	up	Hyper-up
*mmadhca*	UTR3	1.29	up	2.19	up	
*zgc:110699*	UTR3	1.32	up	1.07	up	
*tmem119b*	UTR3	1.24	up	1.01	up	
*zranb1b*	exonic	2.44	up	1.07	up	
*sh3d19*	exonic	2.64	up	1.10	up	
*ctps1b*	UTR3	1.10	up	1.66	up	
*dock4b*	UTR3	1.30	up	1.81	up	
*mnta*	UTR3	1.13	up	1.23	up	
*sialidase-4*	UTR5	2.97	up	1.46	up	
*mtss1la*	UTR3	1.22	up	1.35	up	
*sgms1b*	UTR5	1.97	up	1.25	up	
*bnip3*	UTR3	1.51	up	1.32	up	

**Table 3:** Primer sequences used for qRT-PCR.

Genes		Primer Sequence (5′–3′)	Product Length (bps)	Accession No.
*mettl14*	Forward	TCGGCCGACATGGTACAAAT	120	XM_048197582.1
	Reverse	TGGTCTTGCCAGGGTTGTTT		
*wtap*	Forward	AGAGCTCAAGAGCAGCCAAG	200	XM_048206845.1
	Reverse	GTTCAGAGGCCGTTGAAGGA		
*alkbh5*	Forward	TGCACACAGGCCTCGTATTT	131	XM_048197746.1
	Reverse	AGCCCGGCTCTCTATCTTCA		
*fto*	Forward	ACGGCACAGGAGAACAGAAG	107	XM_048184628.1
	Reverse	GCCTGAAGGATTGTCCTGCT		
*ythdf2*	Forward	CAAAGGGCCCCTCTATCTGC	221	XM_048191909.1
	Reverse	TGGTCACCGGCTTATTCTCG		
*txnipa*	Forward	GAGAACACCTGCTCTCGCAT	168	XM_048201110.1
	Reverse	CACACGAATGCTCTTCCCCT		
*nlrp1*	Forward	ACTCAGCAAAGCAGGAAAAGC	161	XM_048162530.1
	Reverse	AGGTCTCAACGAGGGAAATG		
*nlrp3*	Forward	TGGAGTTGTGTCTCTCCAACG	163	XM_048194926.1
	Reverse	CCTTCCGGACCAGTCCATTC		
*axin2*	Forward	GTCTGAAGCGGGAACAGGAA	121	XM_048190722.1
	Reverse	AAAGGCAGAGAGTGGGATGC		
*bnip3*	Forward	GAGGTGGCAGCAGTCCTAAA	125	XM_048170148.1
	Reverse	ATCACATGGCAGGCTTCCTC		
*mtor*	Forward	GCCTCAAGTTATGCCCACCT	91	XM_048210663.1
	Reverse	CACAACCATCCCCATCTGCT		
*β-actin*	Forward	TCGTCCACCGCAAATGCTTCTA	152	XM_048192430.1
	Reverse	CCGTCACCTTCACCGTTCCAGT		

Abbreviation: methyltransferase 14 (*mettl14*), WT1-associated protein (*wtap*), alkB homolog 5 (*alkbh5*), FTO alpha-ketoglutarate dependent dioxygenase (*fto*), YTH N6-methyladenosine RNA binding protein F2 (*ythdf2*), thioredoxin interacting protein a (*txnipa*), NLR family pyrin domain containing 1 (*nlrp1*), NLR family pyrin domain containing 3 (*nlrp3*), Axis inhibition protein 2 (*axin 2*), BCL2 interacting protein 3 (*bnip3*), mechanistic target of rapamycin kinase (*mtor*), beta-cytoskeletal actin (*β-actin*).

## Data Availability

The authors confirm that the data supporting the findings of this study are available within the manuscript and tables.

## Supplementary Materials

The supporting information can be downloaded at: https://www.mdpi.com/article/10.3390/ijms26094345/s1.
